# Better Working Memory and Motor Inhibition in Children Who Delayed Gratification

**DOI:** 10.3389/fpsyg.2016.01098

**Published:** 2016-07-21

**Authors:** Junhong Yu, Chi-Ming Kam, Tatia M. C. Lee

**Affiliations:** ^1^Laboratory of Neuropsychology, The University of Hong KongHong Kong, China; ^2^Department of Psychology, Institute of Clinical Neuropsychology, The University of Hong KongHong Kong, China; ^3^The State Key Laboratory of Brain and Cognitive Sciences, The University of Hong KongHong Kong, China

**Keywords:** delayed gratification, executive functions, working memory, cognitive inhibition, motor inhibition, preschooler

## Abstract

**Background:** Despite the extensive research on delayed gratification over the past few decades, the neurocognitive processes that subserve delayed gratification remains unclear. As an exploratory step in studying these processes, the present study aims to describe the executive function profiles of children who were successful at delaying gratification and those who were not.

**Methods:** A total of 138 kindergarten students (65 males, 73 females; *M*_age_ = 44 months, *SD* = 3.5; age range = 37–53 months) were administered a delayed gratification task, a 1-back test, a Day/night Stroop test and a Go/no-go test. The outcome measures of these tests were then analyzed between groups using a Multivariate Analysis of Variance, and subsequently a Multivariate Analysis of Covariance incorporating age as a covariate.

**Results:** Children who were successful in delaying gratification were significantly older and had significantly better outcomes in the 1-back test and go/no-go test. With the exception of the number of hits in the go/no-go test, all other group differences remained significant after controlling for age.

**Conclusion:** Children who were successful in delaying gratification showed better working memory and motor inhibition relative to those who failed the delayed gratification task. The implications of these findings are discussed.

## Introduction

Delayed gratification refers to the process of forgoing immediate or short-term rewards to achieve to larger-valued goals in the longer term. Delayed gratification ability was traditionally assessed in young children with the “Marshmallow Test” wherein participants were presented with a marshmallow and were given a choice to eat it or wait for a certain period of time without eating it, such that they could have two marshmallows eventually (Mischel et al., 2011). It has been used as a measure of effortful control and executive function in many studies (Zhou et al., 2012). The latter refers to a set of “higher level” cognitive functions that regulate and controls the “lower level” cognitive processes and goal-directed behaviors (Alvarez and Emory, 2006). In the developmental psychology literature, delayed gratification is often characterized as a “hot” executive function involve in regulating one's emotion in order to accomplish an emotionally arousing task, in contrast to a “cool” executive function that is involved in accomplishing an emotionally-neutral task (Hongwanishkul et al., 2005). The “hot” aspect of delayed gratification is consistent with experimental evidence that suggests being in a bad mood increases one's desire to seek immediate gratification to make themselves feel better (Tice et al., 2001). It is not simply the regulation of negative emotions that affects delayed gratification outcomes, as children who were induced to experienced pride had significantly poorer delay gratification outcomes relative to a control group (Shimoni et al., 2016). This ability to delay gratification in young children have been shown to predict many positive outcomes. For instance, pre-schoolers who were able to delay gratification for a longer period of time had higher levels of resilience, better academic, and social competency, and planning ability in their adolescence (Mischel et al., 1988). Recent research have linked poor delayed gratification in young children to poor eating self regulation, specifically in eating in absence of hunger (Hughes et al., 2015) and behavioral problems (Willoughby et al., 2011; Kim et al., 2012).

Delayed gratification has been theorized to be a complex self-regulatory process involving different cognitive components (Mischel et al., 1989). Early studies supported such a conception by demonstrating the effects of various cognitive manipulations such as distractions and cognitive appraisals on the outcome of delayed gratification tasks (Mischel et al., 1972; Rodriguez et al., 1989). However, the specific cognitive processes that contribute to the delay of gratification remain unclear (Mischel et al., 2011). Specifically, Mischel et al. (2011) proposed a few cognitive processes relating to delayed gratification that need further clarification. These included cognitive inhibition which involves blocking the entry of irrelevant information or suppressing irrelevant thoughts, motor inhibition which involves suppressing a dominant response in favor of an alternative response, and working memory which keeps relevant information active such as the task demands and the knowledge of a larger but delayed reward. Furthermore, working memory has been linked to delayed discounting, a cognitive process that is implicated in delayed gratification(Wesley and Bickel, 2014). Delayed discounting describes the down-valuing of a reward with delay to its receipt and lower working memory capacity is related to steeper delay discounting rates(Hinson et al., 2003). Hence, a low working memory ability might explain the inability to delay gratification as the child may excessively discount the value of the delayed reward.

Despite the sound theoretical components of delayed gratification, research that looked into its neurocognitive correlates of delayed gratification has not been particularly useful in understanding the cognitive processes involved in delayed gratification. This is because most of the recent research that examined delayed gratification and its related neurocognitive correlates typically examined “Hot” executive functions as a whole, usually by reducing delay gratification outcomes along with those of other “Hot” tasks into a single latent factor and then relate them to “cool” tasks, reduced in a similar manner. Though, they have found both “Hot” and “Cool” executive functions correlate well with each other (Willoughby et al., 2011; Kim et al., 2012; Zelazo and Carlson, 2012; Zhou et al., 2012), the “Hot” executive functions may not represent a unitary construct. In fact, the different “Hot” tasks may not even be mutually related to each other in an expected manner. For instance Hongwanishkul et al. (2005) reported that two different “Hot” tasks (delayed gratification and gambling task) were related to each other in a negative manner. In view of such unexpected results, they suggested that the construct of “hot” executive function needs to be further refined. Hence, such general approach in studying “Hot” executive functions may not be adequate in understanding delayed gratification. Indeed, from a neurodevelopmental perspective, the cortical thickness and surface area of various frontal lobe regions associated with executive functions do not expand uniformly in early childhood (Lyall et al., 2015). Correspondingly, the different executive functions do not develop coherently as a whole and may take place in different stages(Anderson, 2002). For instance, motor inhibition and impulse control are the first to develop, followed by attention related functions(Klenberg et al., 2001). Hence, there is a need for research to be selective on the executive function processes to study and instead of studying “Hot” executive functions as a whole. In this regard, the research has been scant and inconclusive. One study (Hongwanishkul et al., 2005) which investigated the relationships between executive function components in young children, did not find a significant relationship between delayed gratification and outcomes on a working memory task (i.e., self-ordered pointing task). The authors, however, found a significant association between outcomes on a card sorting task, a measure of cognitive inhibition, and delayed gratification. Unfortunately, this association was no longer statistically significant after chronological age was controlled for. In another study (Carlson et al., 2014), delayed gratification outcomes were found to be significantly related to measures of working memory, and cognitive inhibition after controlling for age. The evidence from studies using different task paradigms other than the traditional “marshmallow test” to assess delayed gratification outcomes in adult participants has been mixed. For instance, a random letter generation task, which was meant to disrupt inhibition ability, did not significantly influence delayed gratification outcomes on a single key impulsivity task when both tasks were performed concurrently (Caswell et al., 2013). However, in another study (Diekhof and Gruber, 2010), patterns of functional connectivity in the brain that was suggestive of a top-down inhibition of immediate reward desires during a sequential forced-choice task, significantly predicted participants success in pursuing a longer term reward. While these different task paradigms may have provided some clues to the neurocognitive substrates of delayed gratification, they were different from the traditional “marshmallow test” and the results may not generalize well to real life scenarios of delayed gratification (Luerssen et al., 2014). For instance, unlike the other tasks commonly used to assess choice for delayed rewards, the “marshmallow tests” requires the participant to sustain that choice and this act of sustaining a choice parallels many real life goals (Reynolds and Schiffbauer, 2005). Hence, without such a requirement there might be some concerns regarding the ecological validity of these studies.

In addition, the mixed findings in the literature may stem from the differences in demographic characteristics of the studied samples. The age of participants in delayed gratification studies is of major significance given that there might be age-related differences in delayed gratification. Specifically, adults are better at delaying gratification relative to young children (Green et al., 1994), possibly as a result of age-related developments in the prefrontal cortex (Shaw et al., 2008). Certainly more work in this area is needed to clarify the cognitive processes involved in delayed gratification among children.

In order to address the gap in the literature, the current report aims to describe the executive function profile of children who passed or failed the delayed gratification task. These profiles have yet to be reported in the literature and would be useful as a preliminary step in identifying certain components of executive function that may be crucial in delayed gratification. To these ends, the current report compares children who were successful at delaying gratification to those who were not on a battery of executive function tests assessing working memory, cognitive, and motor inhibition. Based upon the above studies on delayed gratification as well as “hot” and “cool” executive functions, we hypothesized that children who successfully delayed gratification would perform better on these tasks of executive functioning, as compared to those who were unsuccessful.

## Methods

### Participants

First-year kindergarten students were recruited from eight Cantonese-speaking kindergartens in Hong Kong as part of a parent longitudinal study on the relationship between emotional regulation and executive functions. The studied sample consisted of 138 kindergarten students (65 males, 73 females) after excluding (1) participants with congenital disorders, (2) participants with special health conditions (e.g., developmental delays, sleep problems), and (3) participants with missing or invalid data (*N* = 35). The mean age of these participants was 44 months (*SD* = 3.5; range: 37–53 months).

### Procedures

Ethics approval for the parent study was granted by a University's research ethics committee. Written informed consent was obtained from the participants' parents. The above tests were administered to the participants in their kindergarten classrooms by trained research assistants. In addition, demographic information was collected from the participants' parents via face-to-face interviews in these classrooms. These data collection took place during the baseline period of the longitudinal study. Participants were given a gift upon completion of the tests. Additionally, the parents were remunerated with HK$400 when their children have participated in all four rounds of data collection in the longitudinal study.

### Measures

#### Delayed gratification test

The delayed gratification test used in this study was adapted from Hongwanishkul et al. (2005). During the test, participants were shown three to four treats on the table, and they were told if they waited for some time, they would get all treats. After that, the participant was asked to rate each snack on how desirable they were. They were told that at any point in time, if they decided not to wait they could ring a bell to summon the experimenter. If this happened, the participant would only receive their least desirable treat. Furthermore, if at any point they were found to have consumed the treat before the end of the waiting period, they were not allowed to take the rest of the treats on the table. By the end of 3 min, if the participant did not ring the bell or consume the treat, they would be rewarded with all the treats on the table. Participants were then classified categorically based upon whether they have waited for at least 3 min to be rewarded with all the treats or have failed to do so. Their delay time (in seconds) or amount of time the participant waited before consuming the treat is recorded. Participants who did not consume the treat by the end of 3 min are recorded with a delay time of 180 s. Participants who did not show any interest or desire in consuming any of the treats (*N* = 8; counted among the 35 participants with missing/invalid data) were excluded from the study.

#### 1-back task

A continuous visual spatial 1-back task was modified with fewer spatial locations to suit the age group of our participants (Jaeggi et al., 2011). During each trial, children were presented with a sequence of stimuli appearing at one of four spatial locations (four grid quadrants) on a computer screen, and the positions of the stimuli were randomly determined. Each stimulus would appear for 1500 ms and the interstimulus intervals were controlled by the experimenter with a key press after a verbal response was made by the participant to indicate whether the current stimulus is located at the “same” or “different” location as the previous stimulus. There were six practice trials and 24 test trials. The test consisted of 8 “same” and 16 “different” location trials. The participants were then scored based on the number of correct trials they have completed. Higher scores corresponded to better working memory.

#### Day/night stroop test

The day/night Stroop test (Gerstadt et al., 1994) was used to assess participants' cognitive inhibition. The task consisted of a congruent and an incongruent condition. In each condition the participant was presented with a sequence of 12 pictures—six of them depicted the sun and the other six depicted the moon. In the congruent condition, children were required to say “day” or “night” whenever a picture of the sun or moon was presented respectively. In the incongruent condition, participants were required to say “night” for the picture of the sun and to say “day” for the picture of the moon. In both conditions, the pictures were presented one at a time in a pseudo-random order. Participants were scored based upon the total number of trials they have responded correctly in each condition. In addition, an interference score was calculated by subtracting the incongruent condition score from the congruent condition score. Lower interference scores and higher scores in the incongruent condition suggest better cognitive inhibition.

#### Go/no-go test

A go/no-go test (Durston et al., 2002) was used to assess inhibitory motor control. Participants were instructed to press a button in response to a target stimulus (picture of a popular cartoon figure) presented on the computer screen and to avoid pressing the button when a non-target stimulus (picture of another cartoon figure) was presented. Twenty-four target stimuli and six non-target stimuli were presented. The stimuli were presented one at a time in a random order and their durations were either 2000 or 300 ms. Specifically, 12 target stimuli and three non-target stimuli were presented for 2000 ms, and 12 target stimuli and three non-target stimuli were presented for 300 ms. The inter-stimulus interval was 800 ms (i.e., blank screen for 300 ms followed by a fixation cross for 500 ms). During the test, participants received positive auditory feedback (“Good Job”) for responding to the targets and not responding to the non-targets. In addition, they also received negative visual feedback (a red cross “X” was shown on the screen) when they responded to a non-target. However, no feedback was given for not responding to a target. For each participant, the number of hits (i.e., response during a target), false alarms (i.e., response during a non-target), total accuracy and sensitivity (i.e., d′) were recorded. A low false alarm rate, high accuracy and sensitivity correspond to good response control and inhibition ability.

### Statistical analyses

Group differences in the demographic variables were tested via a Multivariate Analysis of Variance (MANOVA) and Chi-square Tests. Partial person correlation analyses were conducted between delay time and the other task measures controlling for demographic variables if they were found to be significantly different between groups. Group differences in the outcome measures were tested using a MANOVA and subsequently a Multivariate Analysis of Covariance (MANCOVA) to control for demographic variables if they were found to be significantly different between groups. Due to unequal variances and skewed distributions in the data, bootstrapping techniques were used. Bootstrapping is a re-sampling method that does not make any assumptions on the sample's distribution (Chen and Peng, 2015) and is robust to violations of normality and heteroscedasticity (Parra-Frutos, 2014). Bootstrapping was carried out using a bias-corrected approach with 5000 samples to calculate bias-corrected 95% confidence intervals. Effect sizes for the MANOVA and MANCOVA analyses were calculated using partial η^2^. Statistical significance was set at *p* < 0.05. All analyses were carried out using Statistical Package for the Social Sciences (SPSS version 22) software.

## Results

### Differences in demographic characteristics between groups

A total of 35 participants successfully waited for at least 3 min, while 103 other participants failed to do so. The demographics characteristics of these two groups of participants are shown in Table 1. Participants who waited were slightly, though significantly older than those who did not wait (*p* = 0.012). There were no significant differences in the number of siblings, distribution of genders as well as income groups between these two groups.

**Table 1:** **Participants' characteristics**.

	**Waited (*N* = 35)**	**Did not wait (*N* = 103)**	**Between group comparison**	
			***F***	**χ^2^**
Mean age in months (*SD*)	45.5 (3.5)	43.8 (3.4)	6.55^*^	
**GENDER**				
Male	14	52		1.39
Female	23	54		
Mean no. of siblings (SD)	1.1 (4.0)	0.9 (1.0)	0.64	
**FAMILY INCOME**				
HK$9999 or below	1	6		2.36
HK$10,000–20,999	10	39		
HK$21,000–33,299	11	30		
HK$33,300 or above	15	31		

SD, Standard Deviation.

* p < 0.05.

### Differences in executive functions test scores between groups

The MANOVA on the executive functions measures revealed significant group differences in the 1-back test and all four outcome measures of the go/no-go test (*p*s ≤ 0.034). These differences were associated with small to moderate effect sizes (0.03 ≤ Partial η^2^ ≤ 0.09). Specifically, participants who waited, significantly outperformed those who did not wait in these outcome measures. Given that age was significantly different between groups, a MANCOVA incorporating age as a covariate was carried out. With the exception of the number of hits in the go/no-go test, all other existing group differences remained significant. The MANOVA and MANCOVA did not reveal significant group differences in any of the Stroop test outcomes. The descriptive statistics and results of the MANOVA and MANCOVA are presented in Table 2. Delay time was positively correlated with 1-back test and, Go/no-go accuracy and sensitivity, and negatively correlated with Go/no-go false alarm. The correlation matrix of delay time and the other outcome measures is shown in Table 3.

**Table 2:** **Descriptive statistics, MANOVA, and MANCOVA on measures of executive function ability between groups**.

	**Waited**		**Did not wait**		**MANOVA**			MANCOVA^*^		
	**Mean**	***SD***	**Mean**	***SD***	***F***	***p***	**Partial η^2^**	***F***	***p***	**Partial η^2^**
1-Back test	21.58	2.75	17.21	7.14	12.77	<0.001	0.085	8.28	0.005	0.057
**STROOP TEST**										
Congruent	11.75	0.69	11.64	0.84	0.49	0.519	0.004	0.13	0.716	0.001
Incongruent	10.53	2.31	9.68	2.74	2.75	0.122	0.020	1.90	0.171	0.014
Interference	1.26	2.01	1.90	2.60	1.78	0.18	0.013	1.52	0.219	0.011
**GO/NO-GO**										
Hits	22.61	1.71	21.30	3.51	4.61	0.034	0.033	2.72	0.101	0.020
False alarm	0.33	0.68	1.18	1.65	8.96	0.003	0.061	5.93	0.016	0.042
Accuracy (%)	74.26	6.31	67.06	14.14	8.70	0.004	0.060	5.46	0.021	0.039
Sensitivity (d′)	0.89	0.70	0.02	1.59	10.16	0.002	0.069	6.16	0.014	0.043

SD, Standard Deviation.

* Age in months was included as covariates.

**Table 3:** **Partial correlation among task measures and delay time controlling for age in months**.

	**Delay time**	**1-Back test**	**Stroop test: congruent**	**Stroop test: incongruent**	**Stroop test: interference**	**Go/no-go: hits**	**Go/no-go: alarm**	**Go/no-go: accuracy (%)**	**Go/no-go: sensitivity (d′)**
Delay time	–								
1-Back test	0.188^*^	–							
Stroop test: congruent	0.092	0.233^**^	–						
Stroop test: incongruent	−0.068	0.090	0.313^***^	–					
Stroop test: interference	0.118	0.217^*^	0.953^***^	0.012	–				
Go/no-go: hits	0.104	0.121	−0.062	0.252^**^	−0.145	–			
Go/no-go: false alarm	−0.209^*^	−0.195^*^	−0.093	−0.071	−0.075	−0.240^**^	–		
Go/no-go: accuracy (%)	0.239^**^	0.209^*^	0.042	0.031	0.035	0.509^***^	−0.938^***^	–	
Go/no-go: sensitivity (d′)	0.243^**^	0.204^**^	0.004	0.001	0.004	0.658^***^	−0.831^***^	0.972^***^	–

* p < 0.05,

** p < 0.01,

*** p < 0.001.

## Discussion

The present study sought to investigate if there are differences in the profile of executive function abilities between children who were able to delay their gratification, and those who were not. In the current report, children who delayed their gratification demonstrated superior working memory and motor inhibition relative to those who have failed to delay their gratification.

The superior working memory observed in children who waited, may hint at a significant involvement of working memory in delayed gratification. This however is inconsistent with a previous study that had failed to obtain a significant correlation between with working memory and delayed gratification outcomes (Hongwanishkul et al., 2005). Unlike the present study, the working memory measure used in the previous study specifically assessed for working memory span. The cognitive demands of our 1-back test were somewhat different compared to working memory span tests. In addition to storage capacity, the 1-back test requires the working memory to be continuously updated with relevant information, while simultaneously discarding irrelevant information (Conway et al., 2005). This, relative to a working memory span test, would more closely mirror the process of tracking relevant long-term goals while discarding unwanted information, that is hypothesized to be crucial in delayed gratification (Mischel et al., 2011). Our findings taken together with those of Hongwanishkul et al. (2005) may hint to the involvement of this tracking and discarding process involved in the working memory function that is associated with delayed gratification.

Our results relating to inhibition may suggest a distinction between cognitive and motor inhibition in relation to delayed gratification outcomes. The data showed that motor inhibition outcomes were significantly different between children who waited and those who did not, while cognitive inhibition outcomes did not differ between these two groups of children. Consequently, these findings suggest that motor inhibition may be the more important inhibitory process involved in delayed gratification. This is somewhat inconsistent with previous findings. For instance, a letter generation task which was demonstrated to impair motor inhibitory performance on a stop signal task, did not impair delayed gratification outcomes on the single key impulsivity task (Caswell et al., 2013). On the contrary, cognitive inhibition, as suggested by task-related functional connectivity between the anteroventral prefrontal cortex and nucleus accumbens, was found to be related to delayed gratification outcomes on a sequential forced-choice task (Diekhof and Gruber, 2010). Perhaps, these inconsistent findings could be explained by the different task paradigms employed across studies which may involve somewhat different cognitive demands. It is possible that our task, which mirrors closely to the original delayed gratification test, taps more heavily on motor inhibitory processes as compared to those of cognitive inhibition. Future replication studies may consider using a larger sample of children and a variety of delayed gratification measures to clarify upon these explanations.

Previous research has noted that children from lower socioeconomic classes have underperformed on delayed gratification outcomes relative to their higher socioeconomic class counterparts (Evans and English, 2002; Raver et al., 2011). Though it is not the objective of this study to investigate how exactly are demographic factors linked to delayed gratification outcomes, it is interesting that we did not find a significant income group effect on delay gratification outcomes. Given that the previous research was conducted on primarily on western populations, we speculate that the emphasis on self-control in the current Asian culture (Lan et al., 2011) may have mitigated the effects of income on delayed gratification outcomes. Future research may want to clarify on such a speculation.

The findings of the current report are subjected to a few limitations. Firstly, the cross-sectional nature of this study does not allow causal inferences relating to the relationship between executive functions and delayed gratification. Secondly, the relatively small sample of children who were successful at delaying gratification in this study may limit the generalization of its findings to the general population.

The current report is the first to describe the executive function profiles of children with respect to their performance on a delayed gratification task. As our results indicated, children who were unsuccessful in passing the task had underperformed their successful counterparts in measures of working memory and motor inhibition. We speculate that these children who were weak in delaying their gratification may benefit from interventions that specifically target their working memory and motor inhibition abilities. Evidence from intervention studies has been consistent this speculation. For instance, children who took part in an intervention consisting of small group game activities that specifically targeted executive functioning, such as working memory and inhibitory control, were found have significantly better post-intervention outcomes in a delayed gratification task, compared to their counterparts in the control group (Traverso et al., 2015). This, taken together with those of the current report, highlight the possibility to enhance, or if not support or compensate for certain executive function in children who are weak in delaying their gratifications. The importance of improving their delayed gratification cannot be understated, considering the long-term implications of delayed gratification in academic, psychosocial (Mischel et al., 1988) and health-related outcomes (Schlam et al., 2013).

## Author contributions

TL and CK designed the study. CK collected the data. JY analyzed the data and wrote the manuscript. CK and TL contributed to the writing and critically reviewed the draft of the manuscript by JY.

### Conflict of interest statement

The authors declare that the research was conducted in the absence of any commercial or financial relationships that could be construed as a potential conflict of interest.
